# Growth Parameters and Mortality Rates Estimated for Seven Data-Deficient Fishes from the Azores Based on Length-Frequency Data

**DOI:** 10.3390/life12060778

**Published:** 2022-05-24

**Authors:** Régis Santos, Ualerson Iran Peixoto, Wendell Medeiros-Leal, Ana Novoa-Pabon, Mário Pinho

**Affiliations:** 1 Okeanos—UAc Instituto de Investigação em Ciências do Mar, Universidade dos Açores, Rua Prof. Dr. Frederico Machado 4, 9900-138 Horta, Portugal; ualerson.ip.silva@uac.pt (U.I.P.); wendell.mm.silva@uac.pt (W.M.-L.); ana.mn.pabon@uac.pt (A.N.-P.); mario.rr.pinho@uac.pt (M.P.); 2 IMAR Instituto do Mar, Universidade dos Açores, Rua Prof. Dr. Frederico Machado 4, 9901-862 Horta, Portugal

**Keywords:** demersal, commercial fish, data-limited, life history, size-based method, Northeast Atlantic

## Abstract

Given the scarcity of information suitable for stock assessments, the growth and mortality of seven exploited marine fishes in Azorean waters were estimated based on length-frequency data. The studied species were *Trachurus picturatus*, *Sparisoma cretense*, *Scomber colias*, *Scorpaena scrofa*, *Serranus atricauda*, *Seriola* spp. and *Aphanopus carbo*. The growth parameters *L_∞_* (cm), *k* (year^−1^) and ϕ’ estimated through the ELEFAN_GA_boot routine were set at 55.87, 0.08 and 2.39 for *T. picturatus*; 55.03, 0.11 and 2.53 for *S. cretense*; 55.93, 0.18 and 2.76 for *S. colias*; 61.11, 0.11 and 2.61 for *S. scrofa*; 52.10, 0.12 and 2.51 for *S. atricauda*; 107.33, 0.12 and 3.18 for *Seriola* spp.; and 133.16, 0.09 and 3.19 for *A. carbo*; respectively. The total mortality rate estimated using the length–converted catch curve method was 0.22, 0.35, 0.58, 0.32, 0.31, 0.39 and 0.22 year^−1^; the natural mortality included 0.15, 0.20, 0.30, 0.20, 0.21, 0.21 and 0.16 year^−1^; and fishing mortality rate 0.07, 0.15, 0.28, 0.12, 0.10, 0.18 and 0.06 year^−1^, respectively, for the species mentioned. The relatively large sizes and slow growth with a low natural mortality rate indicate a high vulnerability to overfishing. Therefore, assessment and management initiatives are highly encouraged to ensure the sustainability of the resources.

## 1. Introduction

Knowledge of fish population dynamics (e.g., recruitment, growth and mortality) is essential for improving stock assessment and fisheries management [1,2]. Failure to manage fish populations efficiently may have devastating consequences for biodiversity as well as the livelihoods and socioeconomic situations of millions of people who rely heavily on these resources. Management strategies that successfully conserve the stocks and maximize sustainable yields are based on sufficient knowledge to understand the population dynamics and infer causal linkages between management measures (e.g., harvest control rules) and a fish population [3,4].

Growth parameters and mortality rates of fishes are crucial inputs for stock assessments. This is because they provide valuable information on the variation of fish size over time and the decline in population biomass due to fishing and/or natural causes [1,5]. Traditional methods for determining the growth parameters include direct readings of rigid structures (e.g., otoliths, spines and vertebrae) to estimate the age of fish or indirect estimations based on length distribution data over time [1]. While well-conducted growth studies using otoliths, scales or other rigid structures should produce more accurate growth estimates than studies using solely length-frequency data, length-based approaches are highly desirable, mainly when age data collection is resource-demanding or accurate aging is not possible [6,7,8]. Mortality estimates may be determined by assessing changes in the relative abundance of a certain group or age class of fish in the age-composition data [9]. As an alternative to using age-composition data to predict mortality, a combination of length-composition and growth curve data for the species can then be used [9].

To date, information on the growth and mortality of blue jack mackerel *Trachurus picturatus*, parrotfish *Sparisoma cretense*, Atlantic chub mackerel *Scomber colias*, red scorpionfish *Scorpaena scrofa*, blacktail comber *Serranus atricauda*, amberjacks nei *Seriola* spp., and black scabbardfish *Aphanopus carbo* is still scarce [10,11,12,13,14,15,16,17,18,19,20,21,22,23,24,25,26,27,28,29,30,31] or nonexistent. Traditionally, these species have been caught by small-scale communities that depend on the multi-species catch in nearshore ecosystems to maintain their livelihoods and food security. In the Azores (from 36° to 40° N and from 24° to 32° W), *T. picturatus*, *S. cretense*, *S. colias*, *S. scrofa*, *S. atricauda*, *Seriola* spp. (as *S. dumerili* and *S. rivoliana* are landed in the ports of the region due to their morphological similarity) and *A. carbo* were recently identified as priority species for assessment and management under the European Union Marine Strategy Framework Directive (MSFD; Descriptor 3) and United Nations Sustainable Development Goal 14 (SDG; Indicator 14.4.1) because of their economic, ecological and socio-cultural relevance [32].

*Trachurus picturatus* and *S. colias* are mostly caught by a small coastal fleet that operates with several types of surface nets in the Azores, the most important being the boat-operated purse-seine and lift net. These species are also caught by bottom longlines and handlines, but not as target species [10]. *Trachurus picturatus* and *S. colias* are used for local human consumption or as live bait in the tuna fishery, and during the last years, official landings have averaged 850 t and 300 t per year, respectively [32]. *Sparisoma cretense* and *S. atricauda* are targeted by coastal fisheries using gillnets and hook and lines, with landings of 210 t and 60 t on average per year [10,32]. *Scorpaena scrofa* and *Seriola* spp. are mostly caught by a small-scale bottom longline fishery, whose vessels (mostly less than 12 m in length) operate throughout the year around the islands and seamounts [10]. *Aphanopus carbo* is mainly targeted by a drifting longline fishery that operates in deep waters [32]. Official landings of these last three species have ranged from 30 to 100 t per year [32]. As management measures to reduce fishing impacts on these resources, fishing area restrictions and total allowable catch/quotas have been implemented over the last decades in the Azores [10].

Nevertheless, most of these resources are thought to be intensively exploited [32], and some species have shown a decreasing pattern in their catches [10]. On the other hand, the exploitation and stock status relative to the maximum sustainable yield are unclear due to a deficiency of data on populational aspects [10,32]. Life-history information available concerning growth parameters of those species is restricted to studies of Isidro [33] and Garcia et al. [17] for *T. picturatus*, Carvalho et al. [12] for *S. colias* and Amorim et al. [30] for *S. atricauda*. Studies on the mortality rate have only been undertaken in the Azores for *S. colias* [12] (Table 1).

Due to the relatively low time and financial cost of data collection, some information on length frequency derived from Azorean commercial landings is available for those species. The objective of this study was, therefore, to provide updated and new information on growth parameters and mortality rates of the data-deficient *T. picturatus*, *S. cretense*, *S. colias*, *S. scrofa*, *S. atricauda*, *Seriola* spp. and *A. carbo* species based on length-frequency data to inform stock assessment and improve fisheries management.

## 2. Materials and Methods

### 2.1. Data Collection

Fish species (blue jack mackerel *T. picturatus*, parrotfish *S. cretense*, Atlantic chub mackerel *S. colias*, red scorpionfish *S. scrofa*, blacktail comber *S. atricauda*, amberjacks nei *Seriola* spp. and black scabbardfish *A. carbo*) landed at the Azorean fish markets were sampled under the European Commission’s data collection framework (DCF) [37]. DCF sampling design and protocols were developed in accordance with the recommendations of the International Council for the Exploration of the Sea (ICES) working groups on commercial catches (WGCATCH; https://www.ices.dk/community/groups/Pages/WGCATCH.aspx; accessed on 10 January 2022) and biological parameters (WGBIOP; https://www.ices.dk/community/groups/Pages/WGBIOP.aspx; accessed on 10 January 2022) [38]. For fish species with a forked tail, the fork length (*L_F_*) was obtained instead of the total length (*L_T_*). Table 2 summarizes the number of individuals sampled by species, sampling time and measure of fish size.

### 2.2. Growth Parameters

Growth parameters were estimated through the von Bertalanffy growth function (VBGF) [7] using monthly length-frequency data (2 cm class interval for *S. cretense*, *S. colias*, *S. scrofa* and *S. atricauda*; and 3 cm class interval for *T. picturatus*, *Seriola* spp. and *A. carbo*) obtained from commercial landings. Data from *Seriola* spp. were grouped for this analysis due to a lack of data in some months. As the length data were not separated for males and females, growth parameters were estimated for pooled sexes. The original VBGF model [7] was altered to remove theoretical age at length zero (*t*_0_) as follows:

*L_t_* = *L_∞_* (1 − e^−*k*(*t*)^)

where *L_t_* is the fish length (cm) at age *t* (year), *L_∞_* is the asymptotic length (cm), i.e., the length that the fish of a population would reach if they were to grow indefinitely, and *k* is the growth coefficient that expresses the rate (year^−1^) at which the asymptotic length is approached. The asymptotic length (*L_∞_*), growth coefficient (*k*) and growth performance index (ϕ′) were computed by electronic length-frequency analysis using a bootstrapped method with a genetic algorithm (ELEFAN_GA_boot; [39]) within the *TropFishR* [40,41] and *fishboot* [39,42] packages in R [43]. Bootstrap experiments based on 1000 resamples.

### 2.3. Mortality Rates

The total mortality rate (*Z*; year^−1^) was estimated based on the linearized length–converted catch curve [44] within the *TropFishR* R package [40,41]. For this, it was assumed that the fisheries dynamics associated with each of the stocks analyzed remained reasonably steady during the chosen period. The natural mortality (*M*; year^−1^) was calculated as the average value of natural mortality assessed using different methods [45,46,47,48,49,50,51,52,53,54,55,56,57] (Table 3). The mean annual water temperature (T) was set at 18 °C following the mean annual water temperature of the study area [58,59,60]. Fishing mortality (*F*; year^−1^) was obtained from the relationship: *F = Z − M*.

## 3. Results

### 3.1. Length Distribution

**Figure 1:**
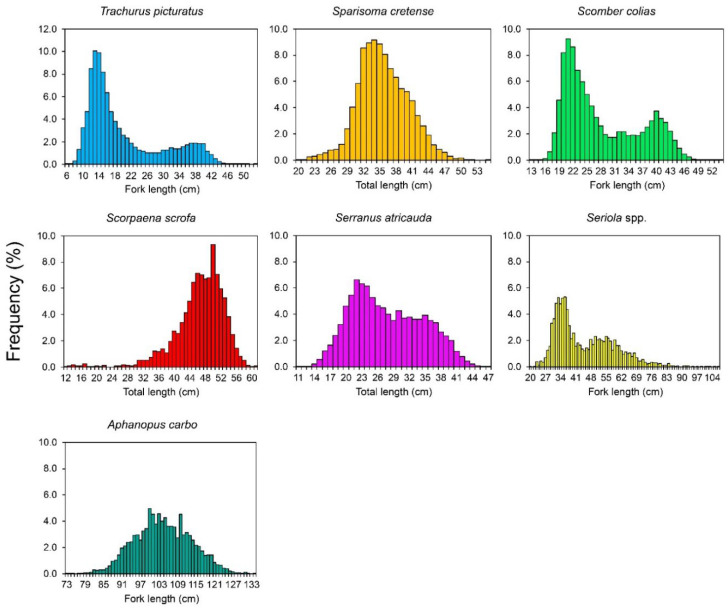
Length-frequency distributions of blue jack mackerel *Trachurus picturatus*, parrotfish *Sparisoma cretense*, Atlantic chub mackerel *Scomber colias*, red scorpionfish *Scorpaena scrofa*, blacktail comber *Serranus atricauda*, amberjacks nei *Seriola* spp. and black scabbardfish *Aphanopus carbo* collected from commercial landings in the Azores between 1990 and 2017 (species-specific sampling periods are shown in Table 2).

The size range of individuals varied from 6 to 53 cm fork length (*L_F_*; mean length ± standard deviation: 24.04 ± 10.8) for *Trachurus picturatus*, from 20 to 55 cm total length (*L_T_*; 36.48 ± 5.50) for *Sparisoma cretense*, from 13 to 54 cm *L_F_* (30.89 ± 8.68) for *Scomber colias*, from 12 to 61 cm *L_T_* (46.85 ± 6.03) for *Scorpaena scrofa*, from 11 to 47 cm *L_T_* (30.25 ± 6.78) for *Serranus atricauda*, from 20 to 107 cm *L_F_* (49.22 ± 14.58) for *Seriola* spp. and from 73 to 134 cm *L_F_* (104.67 ± 10.53) for *Aphanopus carbo* (Figure 1).

### 3.2. Growth Parameters

**Figure 2:**
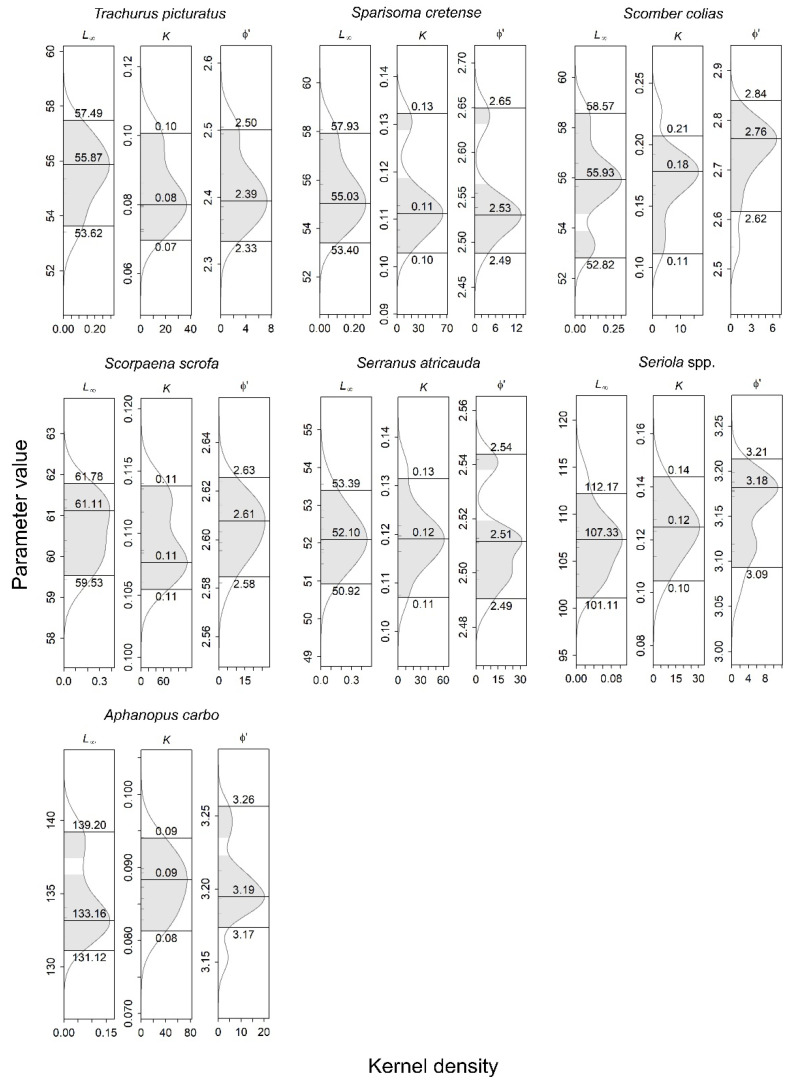
Estimates of asymptotic length (*L_∞_*; cm), growth rate coefficient (*k*; year^−1^), and growth performance index (ϕ’) with 0.95 confidence intervals for blue jack mackerel *Trachurus picturatus*, parrotfish *Sparisoma cretense*, Atlantic chub mackerel *Scomber colias*, red scorpionfish *Scorpaena scrofa*, blacktail comber *Serranus atricauda*, amberjacks nei *Seriola* spp. and black scabbardfish *Aphanopus carbo* in the Azores between 1990 and 2017 (species-specific sampling periods are shown in Table 2). *y*-axis—growth parameter value; *x*-axis—kernel density estimate; horizontal lines—location of the mode (maximum density peak) and the lower and upper 95% quantiles of the univariate kernel density distributions.

For growth parameters, the asymptotic lengths (*L_∞_*) were 55.87, 55.03, 55.93, 61.11, 52.10, 107.33 and 133.16 cm for *T. picturatus*, *S. cretense*, *S. colias*, *S. scrofa*, *S. atricauda*, *Seriola* spp. and *A. carbo*, respectively. The estimated growth coefficients (*k*) were 0.08, 0.11, 0.18, 0.11, 0.12, 0.12 and 0.09 year^−1^, respectively, while the calculated growth performance indices (ϕ′) were 2.39, 2.53, 2.76, 2.61, 2.51, 3.18 and 3.19, respectively, for the same species mentioned. The estimated growth parameters with their 0.95 confidence intervals are shown in Figure 2.

### 3.3. Mortality Rates

**Figure 3:**
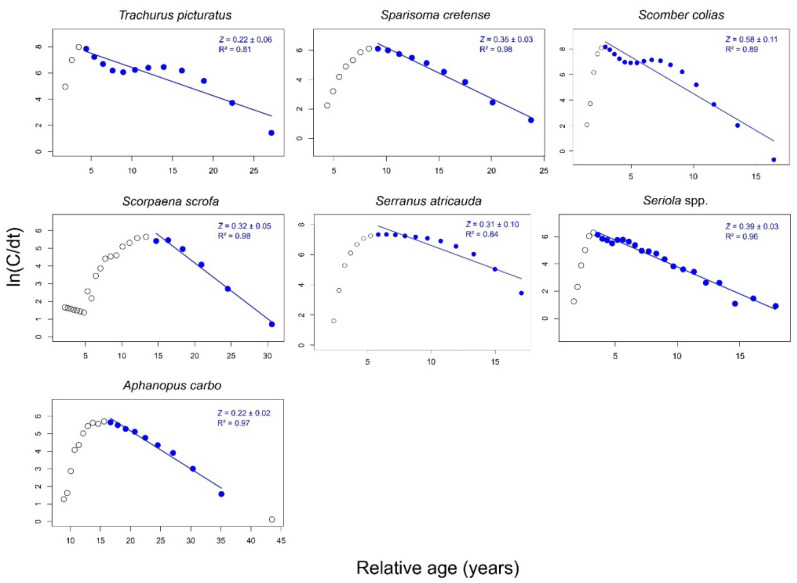
Estimates of total mortality rate (*Z*) from the linearized length–converted catch curve method for blue jack mackerel *Trachurus picturatus*, parrotfish *Sparisoma cretense*, Atlantic chub mackerel *Scomber colias*, red scorpionfish *Scorpaena scrofa*, blacktail comber *Serranus atricauda*, amberjacks nei *Seriola* spp. and black scabbardfish *Aphanopus carbo* in the Azores between 1990 and 2017 (species-specific sampling periods are shown in Table 2).

The total mortality rate (*Z*; mean value ± 0.95 confidence interval) estimated from the linearized length–converted catch curve method was 0.22 ± 0.06, 0.35 ± 0.03, 0.58 ± 0.11, 0.32 ± 0.05, 0.31 ± 0.10, 0.39 ± 0.03 and 0.22 ± 0.02 year^−1^ for *T. picturatus*, *S. cretense*, *S. colias*, *S. scrofa*, *S. atricauda*, *Seriola* spp. and *A. carbo*, respectively (Figure 3). The mean values of natural mortality (*M*) were 0.15, 0.20, 0.30, 0.20, 0.21, 0.21 and 0.16 year^−1^ (Table 3); hence, the fishing mortality rate (*F*) was computed as 0.07, 0.15, 0.28, 0.12, 0.10, 0.18 and 0.06 year^−1^, respectively, for the same species mentioned.

## 4. Discussion

In general, the current investigation indicates a favorable environment for reaching the maximum length compared to all existing records. The maximum *L_F_* of 53 cm for *T. picturatus* and the maximum *L_T_* of 47 cm for *S. atricauda* observed in the present study were close to the maximum *L_F_* of 54 cm for *T. picturatus* observed by Garcia et al. [17] and the maximum *L_T_* of 46 cm for *S. atricauda* reported by Rosa et al. [62]. *S. cretense* and *S. scrofa* had maximum *L_T_* of 55 cm and 61 cm, respectively, which were greater than the 52 cm reported by Afonso et al. [63] for *S. cretense* and the 59 cm reported by Rosa et al. [62] for *S. scrofa*. On the other hand, the maximum *L_T_* of 65 cm (60 cm *L_F_*) for *S. colias* [64] and 160 cm (139 cm *L_F_*) for *Seriola* spp. [18] were found in the literature to be greater than the maximum length obtained in this study. The maximum *L_T_* of *A. carbo* ranged between 120 cm [65] and 148 cm [66], implying that the 134 cm *L_F_* recorded in this study was near the maximum value. Due to the scarcity of existing data for *T. picturatus*, *S. cretense*, *S. colias*, *S. scrofa*, *S. atricauda*, *Seriola* spp., and *A. carbo* in the literature, the findings from this study will undoubtedly contribute to the available records.

The growth parameters of fish population dynamics (asymptotic length *L_∞_* and growth coefficient *k*) are critical for stock assessment and fisheries management. Some reported *L_∞_* for *T. picturatus* in Madeira and Canaries (Table 1) were shorter than the present findings. However, an *L_∞_* of 55.9 cm *L_F_* obtained in this study agreed with the values previously estimated for the Azores by Garcia et al. [17] through direct otolith readings (58.3 cm *L_F_*). The variations among these Macaronesian islands are probably due to stock differences in spatial scale [67]. For *S. cretense*, estimates for the Greek coast from scale readings (*L_∞_* = 38.9 cm *L_T_*) were much lower than those observed for the Azores (*L_∞_* = 57.9 cm *L_T_*). Longer *L_∞_* than those reported in the literature for *S. colias* [12,13,16,22,29] and *S. atricauda* [30,31] were also observed in this study. These differences are most likely due to the larger specimens present in this study. On the other hand, *S. scrofa*, *Seriola* spp., and *A. carbo* were found to have smaller *L_∞_* in the Azores compared to other regions in the Atlantic [19,20,21,23,25,26] and Mediterranean [18,35].

Considering the estimated growth coefficients (*k*) for *T. picturatus*, *S. cretense*, *S. colias*, *S. scrofa*, *S. atricauda*, *Seriola* spp. and *A. carbo* calculated in the present study, Sparre and Venema [1] proposed that *k* = 1.0 year^−1^ indicates fast growth, *k* = 0.5 year^−1^ moderate growth and *k* = 0.2 year^−1^ indicates slow growth, suggesting that the seven species described here grow at a relatively slow rate. The estimated *k* values are smaller or approximately equal to those previously observed in the Azores, Madeira and Canaries for *T. picturatus*; Greece for *S. cretense*; Azores, Gulf of Cádiz, Alboran Sea, Adriatic Sea and Mauritania for *S. colias*; Azores and Canaries for *S. atricauda*; NW Atlantic, Gulf of Mexico and Adriatic Sea for *Seriola* spp.; and Canaries and Madeira for *A. carbo* (Table 1). For *S. scrofa*, the estimated *k* was higher than that observed by Shahrani and Shakman [35] on the Libyan coast (Table 1).

Within the same species, variations in *L_∞_* and *k* can be attributed to various causes, including variations in water conditions, food availability, metabolic rate, fishing pressure and pollution [1]. For example, as fishing gear is selective and oriented toward harvesting larger individuals, large individuals may become rare in overexploited fisheries, and a scarcity of these individuals in a given sample will inevitably underestimate growth parameters [68,69]. On the other hand, while analyzing fish growth, the validation of growth parameter estimations often arises due to the reliability of some of the methodologies employed to produce such estimates. In this regard, the growth performance index (ϕ’) can indicate estimation reliability since it has been suggested that ϕ’ values are similar for the same species and genera [39,70,71]. The values of ϕ’ obtained from this study (Figure 2) were close to or within the range of values obtained for stocks of *T. picturatus*, *S. cretense*, *S. colias*, *S. scrofa*, *S. atricauda*, *Seriola* spp. and *A. carbo* from other Atlantic areas and the Mediterranean, which were estimated either by direct or indirect methods (Table 1).

The natural mortalities (year^−1^) estimated in this study (Table 3) were not in agreement with the natural mortalities found for *T. picturatus* in Madeira and Canaries, *S. cretense* on the Greek coast, *S. colias* in the Azores, Adriatic Sea and Mauritania, and *Seriola* spp. in the Adriatic Sea (Table 1). Natural mortality varies with age, density, disease, parasites, food supply, predator abundance, water temperature, sex and size; therefore, observed variances could be related to these factors [72]. However, it is interesting to note that the values of natural mortality of the seven fish species in the Azores indicated lower natural mortality, as the values found in this study were mostly at the lower limit of the modal mortality rate (i.e., 0.20) derived from 175 fish stocks as given by Pauly [52]. No reports of mortality parameters for *S. scrofa*, *S. atricauda*, or *A. carbo* are currently available in the literature.

Natural mortality (*M*) and fishing mortality (*F*) can denote the indication of an overfishing status [5]. The optimal scenario for a population is when fishing mortality equals natural mortality, which means that fishing operations exploit the portion of the population that would otherwise be lost due to natural mortality [73]. With knowledge of these parameters, one can manage the stocks and establish the optimal exploitation rate (i.e., *E* = *F*/(*F* + *M*) = 0.5) that maximizes catch while maintaining the reproductive proportion of the population [73]. Values of *M* and *F* were estimated as 0.15 and 0.07 year^−1^ (*E* = 0.3) for *T. picturatus*, 0.20 and 0.15 year^−1^ (*E* = 0.4) for *S. cretense*, 0.30 and 0.28 year^−1^ (*E* = 0.5) for *S. colias*, 0.20 and 0.12 year^−1^ (*E* = 0.4) for *S. scrofa*, 0.21 and 0.10 year^−1^ (*E* = 0.3) for *S. atricauda*, 0.21 and 0.18 year^−1^ (*E* = 0.5) for *Seriola* spp., and 0.16 and 0.06 year^−1^ (*E* = 0.3) for *A. carbo*. These values suggest that *S. cretense*, *S. colias* and *Seriola* spp. have optimum exploitation rate (*M* ≈ *F*), but *T. picturatus*, *S. scrofa*, *S. atricauda* and *A. carbo* are underexploited (*M* > *F*).

However, the exploitation status suggested from this study should be treated with caution because, as population growth estimates have, in some cases, large confidence intervals (see Figure 2) as well as fishing-related uncertainties, the mortality rate scenario may be of more concern. For *T. picturatus*, *S. colias* and *Seriola* spp., for example, the total mortality estimates may also be biased due to the different fishing gears operating over this stock (surface nets and longlines for *T. picturatus* and *S. colias* [10,32]) or the different species (i.e., *S. dumerili* and *S. rivoliana*) grouped as the same stock (i.e., *Seriola* spp.). This condition may even be associated with the bimodal patterns in length-frequency distribution (Figure 1) and catch curves (Figure 3) observed for these species and implies that mortality estimates should ideally be estimated by fishery separately or, in the case of *Seriola* spp., by species. However, the length of data used in this study did not allow this separation, and it should be re-evaluated to make these analyses possible in the future.

For stock assessment, fisheries experts prefer to use age-composition data acquired by reading rigid structures since these data are more likely to reflect the real age composition of the stock than those produced using a proxy for the age composition obtained using length-composition data. However, measuring the length of a fish is significantly less expensive than determining its age, and most of the biological data acquired for Azorean fish populations are in the form of length composition rather than age composition. Therefore, in the absence of otoliths, length-frequency data may be useful for estimating growth parameters and mortality rates for those data-deficient species. Nevertheless, to reduce imprecision and improve accuracy, it would be desirable to confirm these estimations by comparing the results of the analysis to those produced from age-based approaches. This is highly encouraged to be conducted, at least for blue jack mackerel *T. picturatus*, parrotfish *S. cretense*, Atlantic chub mackerel *S. colias*, red scorpionfish *S. scrofa*, blacktail comber *S. atricauda*, amberjacks nei *Seriola* spp. and black scabbardfish *A. carbo* that are data-deficient and at the same time classified as priority stocks for the region.

Since harvesting of the studied species is not sex-based, stock assessment models should need a single set of population parameters. Nonetheless, the disparities in development between the sexes may require a change in management regulations to limit effort, especially on the target component, which is particularly vulnerable to overexploitation. Future research should therefore investigate these particularities since the dataset used in this study did not allow progress in this way. This is even more important when it involves sequential hermaphroditic species with sexual dimorphism, such as *S. cretense* [27]. Additionally, captures of resources such as *T. picturatus*, *S. cretense*, *S. colias*, *S. atricauda* and *Seriola* spp. are not completely landed since they are also fished for self-consumption or utilized as live bait in tuna fisheries [10,32]. Therefore, samples from the commercial landings may be under-represented, and special attention will need to be given to the sampling design to ensure that representative samples are taken. In addition to that, stock units for all the studied stocks are not clearly defined [10]. As a result, it is impossible to know whether the stocks were fully sampled across their entire range of distribution and dynamic aspects of the population or whether the samples analyzed referred only to a fraction of the population.

Although the results of this study have certain limitations, it adds new information on species for which there is little or no information available. The information reported greatly improve understanding of population structure, mortality and exploitation status of blue jack mackerel *T. picturatus*, parrotfish *S. cretense*, Atlantic chub mackerel *S. colias*, red scorpionfish *S. scrofa*, blacktail comber *S. atricauda*, amberjacks nei *Seriola* spp. and black scabbardfish *A. carbo* and inform additional stock assessment initiatives. Stock assessment is a long-term and dynamic process, and a complete picture of the situation can only be obtained when the conclusions from one analysis are compared with those of a different analysis and the different results are used critically to gauge conclusions, improve the data and thus enable future assessments to be more accurate [74].

## 5. Conclusions

The seven fish species examined (i.e., blue jack mackerel *T. picturatus*, parrotfish *S. cretense*, Atlantic chub mackerel *S. colias*, red scorpionfish *S. scrofa*, blacktail comber *S. atricauda*, amberjacks nei *Seriola* spp. and black scabbardfish *A. carbo*) are economically important food fish, and therefore, the estimated population parameters constitute a landmark in the development of stock assessment studies. This information can be implemented into age-structured or length-based models and allow understanding of the dynamics of marine resources and estimating the sustainable yield of the target population. However, uncertainties regarding the representativeness of commercial landing samples in this study have been raised for some species, particularly those that escape the auction control or are used as live bait in other fisheries. In addition, natural mortality and growth patterns are often associated with the ecosystem, species behavior and fishing pressure. As a result, further study should focus on feeding habits, predation–prey relationships, migration patterns, recruitment and the reliability of the current sampling programs for these species. The latter is critical for integrating different data sources to guarantee that small, medium and large individuals are included in the analyses and prevent misleading results. Finally, studies focused on stock delimitation are essential in the stock assessment process because they will allow to estimate reliable population parameters, develop optimal harvest and monitoring strategies, and propose effective conservation measures.

## Figures and Tables

**Table 1:** Summary of growth and mortality studies of *Trachurus picturatus*, *Sparisoma cretense*, *Scomber colias*, *Scorpaena scrofa*, *Serranus atricauda*, *Seriola* spp. and *Aphanopus carbo*.

	Area	Method	*n*	Size	Growth Parameter			Mortality Rate		
					*L* *_∞_*	*k*	ϕ′	*Z*	*M*	*F*
***T. picturatus***										
Isidro [33]	Azores	DR	516	*L_F_*	52.9	0.20	2.75			
Vasconcelos et al. [11]	Madeira	DR	578	*L_T_*	42.3	0.16	2.46			
Vasconcelos et al. [11]	Madeira	BC	229	*L_T_*	48.3	0.13	2.50			
Garcia et al. [17]	Azores	DR	1420	*L_F_*	58.3	0.09	2.48			
Garcia et al. [17]	Azores	BC	796	*L_F_*	52.9	0.11	2.49			
Jurado-Ruzafa and Santamaria [28]	Canaries	DR	913	*L_T_*	34.9	0.21	2.42		0.40	
Vasconcelos et al. [15]	Madeira	IM	10,713	*L_T_*		0.16		0.75–1.64	0.33	
Neves et al. [34]	Mainland Portugal	BC	376	*L_T_*	46.7	0.15	2.51			
Neves et al. [34]	Mainland Portugal	DR	575	*L_T_*	63.3	0.07	2.45			
***S. cretense***										
Petrakis and Papaconstantinou [27]	Greece	DR	399	*L_T_*	38.9	0.16	2.38	0.81	0.38	0.43
***S. colias***										
Carvalho et al. [12]	Azores	DR	349	*L_T_*	57.5	0.20	2.82		0.19	
Velasco et al. [22]	Gulf of Cádiz	BC	121	*L_T_*	43.0	0.25	2.7			
Velasco et al. [22]	SW Mediterranean	BC	98	*L_T_*	40.0	0.37	2.77			
Čikeš Keč and Zorica [29]	Adriatic Sea	DR	280	*L_F_*	45.3	0.18	2.57	0.91	0.35	0.56
Jurado-Ruzafa et al. [16]	Mauritania	BC	163	*L_T_*	48.4	0.25	2.76		0.47	
Daley and Leaf [13]	Gulf of Mexico	DR	60	*L_T_*	33.6	1.75	3.29			
***S. scrofa***										
Shahrani and Shakman [35]	Libya	DR	94	*L_T_*	116.0	0.05	2.82			
***S. atricauda***										
Tuset et al. [31]	Canaries	DR	406	*L_T_*	43.9	0.16	2.49			
Tuset et al. [31]	Canaries	BC	15	*L_T_*	52.3	0.09	2.39			
Tuset et al. [31]	Canaries	MX	490	*L_T_*	49.5	0.11	2.43			
Amorim et al. [30]	Azores	EF	-	*L_T_*	41.4	0.26	2.65			
***Seriola*** **spp.**										
Manooch and Potts [25]	SE U.S.A.	BC	190	*L_F_*	151.4	0.12	3.44	0.60–0.65		
Manooch and Potts [26]	Gulf of Mexico	BC	291	*L_F_*	110.9	0.23	3.45	0.68–0.70		
Thompson et al. [21]	Gulf of Mexico	DR	552	*L_F_*	138.9	0.25	3.68			
Kožul et al. [18]	Adriatic Sea	DR	298	*L_T_*	174.6	0.19	3.76	0.41	0.3	0.11
Harris et al. [23]	SE U.S.A.	DR	1985	*L_F_*	124.2	0.28	3.64			
***A. carbo***										
Morales-Nin and Sena-Carvalho [19]	Madeira	DR	649	*L_T_*	138.6	0.25	3.68			
Pajuelo et al. [20]	Canaries	DR	298	*L_T_*	147.7	0.20	3.64			
Vieira et al. [36]	Madeira	BC	436	*L_T_*	146.1–158.6	0.12–0.15	3.48–3.49			
Vieira et al. [36]	Mainland Portugal	BC	1075	*L_T_*	124.0–135.4	0.17–0.21	3.49–3.50			
Delgado et al. [14]	Madeira	BC	587	*L_T_*	131.9–136.2	0.15–0.17	3.45–3.46			

Notes: DR—direct reading; BC—back-calculation; IM—indirect method; MX—mixing; EF—empirical formula; *n*—number of individuals; *L_T_*—total length; *L_F_*—fork length; *L_∞_*—asymptotic length (cm); *k*—growth coefficient (year^−1^); ϕ′—growth performance index; *Z*—total mortality rate (year^−1^); *M*—natural mortality rate (year^−1^); *F*—fishing mortality rate (year^−1^).

**Table 2:** Sampling period and sample size (*n*) of fish species.

Scientific Name	Common Name	Sampling Period	*n*	Size Measurement
*Trachurus picturatus*	Blue jack mackerel	2010–2017	104,299	*L_F_*
*Sparisoma cretense*	Parrotfish	2010–2017	10,217	*L_T_*
*Scomber colias*	Atlantic chub mackerel	2010–2017	57,723	*L_F_*
*Scorpaena scrofa*	Red scorpionfish	2010–2017	2983	*L_T_*
*Serranus atricauda*	Blacktail comber	2010–2017	31,045	*L_T_*
*Seriola* spp.	Amberjacks nei	1990–2017	3998	*L_F_*
*Aphanopus carbo*	Black scabbardfish	2011–2014	6029	*L_F_*

Notes: *L_F_*—fork length. *L_T_*—total length.

**Table 3:** Estimates of natural mortality (*M*; year^−1^) for blue jack mackerel *Trachurus picturatus*, parrotfish *Sparisoma cretense*, Atlantic chub mackerel *Scomber colias*, red scorpionfish *Scorpaena scrofa*, blacktail comber *Serranus atricauda*, amberjacks nei *Seriola* spp. and black scabbardfish *Aphanopus carbo* from the empirical relationships between the estimated asymptotic length (*L_∞_;* cm) and growth rate coefficient (*k*; year^−1^).

Empirical Formula	Source	Species						
		*Trachurus picturatus*	*Sparisoma cretense*	*Scomber colias*	*Scorpaena scrofa*	*Serranus atricauda*	*Seriola* spp.	*Aphanopus carbo*
*M* = 5/*t_max_*	[45]	0.13	0.18	0.30	0.18	0.20	0.20	0.15
*M* = 2.996/*t_max_*	[46]	0.08	0.11	0.18	0.11	0.12	0.12	0.09
*M* = 3/*t_max_*	[50]	0.08	0.11	0.18	0.11	0.12	0.12	0.09
*M* = 3 *k*/(exp(0.38 *t_max_* × *k* − 1))	[51]	0.21	0.29	0.47	0.29	0.31	0.31	0.24
*M* = exp(− 0.0066 − 0.279 log(*L_∞_*) + 0.6543 log(*k*) + 0.4634 log(T))	[52]	0.53	0.58	0.67	0.58	0.60	0.55	0.50
*M* = 3/*t_max_*	[53]	0.08	0.11	0.18	0.11	0.12	0.12	0.09
*M* = 4.6/*t_max_*	[54]	0.12	0.17	0.28	0.17	0.18	0.18	0.14
*M* = 1.0661 *L_∞_*^− 0.1172^ × *k* ^0.5092^	[55]	0.18	0.22	0.28	0.21	0.23	0.21	0.18
*M* = − 0.1778 + 3.1687 *k*	[56]	0.08	0.17	0.39	0.17	0.20	0.20	0.11
*M* = 1.6 *k*	[57]	0.13	0.18	0.29	0.18	0.19	0.19	0.14
*M* = 1.5 *k*	[57]	0.12	0.17	0.27	0.17	0.18	0.18	0.14
*M* = 1.4 *k*	[47]	0.11	0.15	0.25	0.15	0.17	0.17	0.13
*M* = 4.22/*t_max_*	[48]	0.11	0.15	0.25	0.15	0.17	0.17	0.13
*M* = 4.22/*t_max_*	[49]	0.11	0.15	0.25	0.15	0.17	0.17	0.13
Mean value		0.15	0.20	0.30	0.20	0.21	0.21	0.16

Notes: *t_max_*—the approximate maximum age (years) = 3/*k* [61]; T—water temperature (°C).

## Data Availability

The data underlying this article will be shared upon reasonable request to the corresponding author.
